# Disruption of Photomorphogenesis Leads to Abnormal Chloroplast Development and Leaf Variegation in *Camellia sinensis*

**DOI:** 10.3389/fpls.2021.720800

**Published:** 2021-09-09

**Authors:** Xizhi Gao, Chenyu Zhang, Cui Lu, Minghan Wang, Nianci Xie, Jianjiao Chen, Yunfei Li, Jiahao Chen, Chengwen Shen

**Affiliations:** ^1^Key Laboratory of Tea Science of Ministry of Education, Hunan Agricultural University, Changsha, China; ^2^National Research Center of Engineering and Technology for Utilization of Botanical Functional Ingredients, Hunan Agricultural University, Changsha, China; ^3^Co-Innovation Center of Education Ministry for Utilization of Botanical Functional Ingredients, Hunan Agricultural University, Changsha, China; ^4^Tea Research Institution, Chinese Academy of Agricultural Sciences, Hangzhou, China; ^5^Institution of Genomics and Bioinformatics, South China Agricultural University, Guangzhou, China

**Keywords:** *Camellia sinensis*, variegated, transcriptome sequencing, chloroplast development, photomorphogenesis

## Abstract

*Camellia sinensis* cv. ‘Yanlingyinbiancha’ is a leaf-variegated mutant with stable genetic traits. The current study aimed to reveal the differences between its albino and green tissues, and the molecular mechanism underlying the variegation. Anatomic analysis showed the chloroplasts of albino tissues to have no intact lamellar structure. Photosynthetic pigment in albino tissues was significantly lower than that in green tissues, whereas all catechin components were more abundant in the former. Transcriptome analysis revealed most differentially expressed genes involved in the biosynthesis of photosynthetic pigment, photosynthesis, and energy metabolism to be downregulated in albino tissues while most of those participating in flavonoid metabolism were upregulated. In addition, it was found cryptochrome 1 (CRY1) and phytochrome B (PHYB) genes that encode blue and red light photoreceptors to be downregulated. These photoreceptors mediate chloroplast protein gene expression, chloroplast protein import and photosynthetic pigment biosynthesis. Simultaneously, SUS gene, which was upregulated in albino tissues, encodes sucrose synthase considered a biochemical marker for sink strength. Collectively, we arrived to the following conclusions: (1) repression of the biosynthesis of photosynthetic pigment causes albinism; (2) destruction of photoreceptors in albino tissues suppresses photomorphogenesis, leading to abnormal chloroplast development; (3) albino tissues receive sucrose from the green tissues and decompose their own storage substances to obtain the energy needed for survival; and (4) UV-B signal and brassinosteroids promote flavonoid biosynthesis.

## Introduction

The tea plant [*Camellia sinensis* (L.) O. Kuntze] is an important economic crop that is cultivated worldwide. Leaf-color variation in tea plants has been extensively studied. Till date, lots of leaf color phenotypes, such as green, white, yellow, purple, and variegated, have been found in tea plants (Li et al., 2016a; Song et al., 2017; Shen et al., 2018). Leaf color is usually determined by pigments, such as chlorophyll, anthocyanins, and carotenoids. In general, the altered gene expression of leaf-color mutants can directly or indirectly affect the synthesis, degradation, content, and proportion of pigment, thereby blocking photosynthesis and causing abnormal leaf color (Zhao et al., 2020). Furthermore, significant differences exist in the content of main biochemical components, such as catechins, amino acids, flavonoids, and other substances, in tea plants with different leaf colors. Albino tea plants include three types of colors: albino, etiolated, and variegation. Compared to normal green tea plants, albino tea plants are precious, due to the special flavor. Albino tea leaves usually had a higher abundance of free amino acids, along with lower levels of catechins and caffeine, compared with green tea leaves (Feng et al., 2014). Change of these metabolites leads to decreased bitterness and astringency, and enhanced umami taste of the albino tea.

Variegation is a common phenotype in higher plants, with both green and white (or yellow) areas on the same leaf. Variegated mutants are important materials for studying the mechanism of development and maintenance of plant plastids (Sakamoto, 2003). Variegated mutants in *Arabidopsis thaliana* have been studied in much detail. The *immutans* (*im*) mutant of Arabidopsis produces reactive oxygen species due to the deletion of plastid terminal oxidase, thereby resulting in photobleaching to form a variegated phenotype (Carol et al., 1999). VAR1 and VAR2 are the main components of the FtsH complex that repairs photodamaged proteins in the thylakoid membrane. Both *var1* and *var2* mutants are susceptible to photodamage under intense light, leading to a yellow variegated phenotype (Sakamoto et al., 2003). While variegation may occur due to several reasons, it will eventually lead to the loss or abnormality of chloroplasts. At present, there are fewer variegated mutants in tea plants, such as ‘Yinghongjiuhao’. Ma et al. found that almost all the proteins mapped in the photosynthetic pathway showed decreased expression in ‘Yinghongjiuhao’ variegated mutant compared to green leaves (Ma et al., 2016).

Light, one of the main environmental factors that determine the biogenesis of chloroplasts, controls gene transcription, chlorophyll biosynthesis, and protein degradation through photomorphogenesis (Pogson and Albrecht, 2011). Photomorphogenesis is the process by which plants grow and develop in response to light signals. This process is mediated by a complex network of photoreceptors, including phytochromes (PHYs), cryptochromes (CRYs), and phototropins (PHOTs) (Han et al., 2007). CONSTITUTIVE PHOTOMORPHOGENIC 1 (COP1), a master negative regulator of light signaling pathway, targets multiple transcription factors of light signaling pathways for repressing photomorphogenesis (Lau and Deng, 2012). Upon irradiation of light, the photoreceptors inactivate COP1 through direct protein–protein interaction and/or nuclear exclusion of COP1, and thereby facilitate photomorphogenesis (Podolec and Ulm, 2018; Xu, 2020). PHYB has also been reported to play an important role in the biosynthesis of chlorophyll and carotenoid, and development of chloroplast membrane (Zhao et al., 2013; Toledo-Ortiz et al., 2014). Photosynthetic pigments are essential for the assembly and protection of photosynthetic complexes, and thereby promoting chloroplast development. CRY has also been confirmed to be involved in inducing the expression of genes related to photosynthesis (Ohgishi et al., 2004). Once the photomorphogenesis is disrupted, photosynthetic pigment biosynthesis and chloroplast development will be blocked, leading to the inhibition of photosynthesis. Subsequently, energy metabolism is also influenced due to insufficient carbon supply. Taken together, photomorphogenesis is likely to play a prominent role in the formation of variegated plants.

A variegated mutant of ‘Yanlingyinbiancha’, with white edge and green main veins, is found in Yanling County, Hunan, China. After multiple generations of asexual reproduction and trial planting, the phenotypic traits have stabilized. In this study, we aimed to conduct transcriptome sequencing of the white and green tissues of ‘Yanlingyinbiancha’ in order to reveal the mechanism underlying variegation.

## Materials and Methods

### Plant Materials

The tea plant [*Camellia sinensis* (L.) O. Kuntze cv. ‘Yanlingyinbiancha’] was cultivated in Yanling County, Hunan, China. In 2017, one bud and two leaves were plucked and washed using ultrapure water. The albino and green tissues were then cut separately with scissors, fixed with liquid nitrogen immediately, and stored in a −80°C refrigerator.

### Transmission Electron Microscopy Analysis

Fresh leaf was sliced into 1 mm^2^ sections, then fixed with 2.5% (v/v) glutaraldehyde for 2 h at 20°C and rinse three times with phosphate buffered solution (PBS) pH 7.4 for 15 min each. Leaf cells were post-fixed with 1% (v/v) OsO4 at 20°C for 5 h and then dehydrated in a series ethanol solutions (30, 50, 70, 80, 90, 95, and 100% for 1 h each). Subsequently, the samples were embedded in prepared 100% Epon-812 and polymerized in oven at 60°C for 48 h. The samples were cut into ultrathin sections (60–80 nm) using an ultramicrotome (EM UC7, Leica Microsystems Inc., Mannheim, Germany), and then stained with uranyl acetate and lead citrate for 30 min. Finally, images acquisition and analysis were obtained with a TEM (HT7700, Hitachi Ltd., Tokyo, Japan).

### Catechin, Caffeine, Gallic Acid, and Theobromine Content

Catechin, CAF, GA, and TB contents were determined by high-performance liquid chromatography. Catechin and CAF contents were determined based on protocols from the National Standards of the People's Republic of China (GB/T 8313-2018 and GB/T 8314-2013). The HPLC system (Waters 590; Waters Corp.) was equipped with a Hypersil ODS 2 C18 column (5 ml, 4.6 mm 250 mm, 35 c) and the detection wavelength was set at 280 nm. Solvents A (2% acetic acid) and B (acetonitrile) were run in a linear gradient for 20 min, from 93 to 55%, and which the flow rate was maintained for 5 min at 1.4 mL min^−1^. Caffeine and catechin were quantitatively determined by comparing the peak areas of samples with known standards.

### Photosynthetic Pigments

The methods used to determine the photosynthetic pigments (chlorophyll a, chlorophyll b, and carotenoids) contents of leaves were as follows: 20 mg of fresh tea leaves were cut into several filaments of ≥20 mm length and ≤1 mm width along the central vein. The strips were placed in 5 mL of 95% ethanol and incubated in the dark for 12 h. The extracts were filtered and analyzed with a UV-2550 spectrophotometer (Shimadzu, Japan), and their absorbance was recorded at the following wavelengths: 665 nm for chlorophyll a, 649 nm for chlorophyll b, and 470 nm for total carotenoids. Calculation formula: Chla (mg/L) = 13.95A_665_ – 6.88A_649_; Chlb (mg/L) = 24.96A_649_ – 7.32A_665_; Car (mg/L) = (1000A_470_ – 2.05Chla – 114.8Chlb)/245.

### Transcriptome Analysis

#### RNA Preparation, Library Construction, and Sequencing

Total RNA of albino and green tissues was extracted using TRIzol reagent (Invitrogen, Carlsbad, CA, USA). RNA integrity was assessed using the RNA 6000 Nano Assay Kit in the Bioanalyzer 2100 system (Agilent Technologies, Santa Clara, CA, USA). A total amount of 1 μg RNA per sample was used as input material for the RNA sample preparations. Briefly, mRNA was purified from total RNA using poly-T oligo-attached magnetic beads. First strand cDNA was synthesized using random hexamer primer and M-MuLV Reverse Transcriptase (RNase H-). Second strand cDNA synthesis was subsequently performed using DNA Polymerase I and RNase H. The library fragments were purified with AMPure XP system (Beckman Coulter, Beverly, USA). Then PCR was performed with Phusion High-Fidelity DNA polymerase, Universal PCR primers and Index (X) Primer. PCR products were purified (AMPure XP system) and library quality was assessed on the Agilent Bioanalyzer 2100 system. At last, the library preparations were sequenced on an Illumina Novaseq platform and 150 bp paired-end reads were generated.

#### Data Analysis

Raw data (raw reads) of fastq format were firstly processed through in-house perl scripts, including removing reads containing adapter, reads containing ploy-N and low quality reads from raw data, and calculating Q20, Q30, and GC content the clean data. The remaining high-quality clean reads were aligned to *C.sinensis* reference genome using Hisat2 (v2.0.5) (Wei et al., 2018). For gene annotation, all UniGenes were searched against the Kyoto Encyclopedia of Genes and Genomes (KEGG) and Gene Ontology (GO) databases by BLAST (E value < 1.0E^−6^).

#### Differential Expression Analysis

featureCounts (v1.5.0-p3) was used to count the reads numbers mapped to each gene. Then, fragments per kilobase per million (FPKM) of each gene was calculated based on the length of the gene and reads count mapped to this gene. Differential expression analysis was performed using the DESeq2 R package (v1.20.0). Genes with an adjusted *P*-value < 0.05 found by DESeq2 were assigned as differentially expressed.

#### GO and KEGG Enrichment Analysis

GO and KEGG enrichment analysis of differentially expressed genes (DEGs) was implemented by the clusterProfiler R package, in which gene length bias was corrected. GO terms/KEGG pathways with corrected *P*-value < 0.05 were considered significantly enriched by differential expressed genes.

### Quantitative Real-Time Polymerase Chain Reaction

Total RNA was extracted using an RNA extraction kit (Tiangen, Beijing, China), and complementary DNA (cDNA) was synthesized using a PrimeScript™ RT Reagent Kit (Takara, Dalian, China). Supplementary Table 1 lists the primer pairs used for qRT-PCR; β-actin was used as the reference gene. The thermal cycling protocol followed the manufacturer's instructions. Three independent biological replicates of each reaction were conducted, and the relative transcript levels of target genes were calculated against those of β-actin using the formula $2_{\text{T}}^{-\Delta \Delta \text{C}}$ (Livak and Schmittgen, 2001).

### Statistical Analysis

Analysis of variance was performed with SPSS software (v22.0, SPSS Inc., Chicago, IL) to determine the least significant differences between different treatments (*p* < 0.05), and Duncan's multiple range test was used to compare the averages. Mean and standard deviation were calculated based on six independent biological replicates per tissue. GraphPad Prism (v8.0.1) was used to process the data and generate the figures.

## Results

### Changes in Ultrastructures and Photosynthetic Pigments

**Figure 1:**
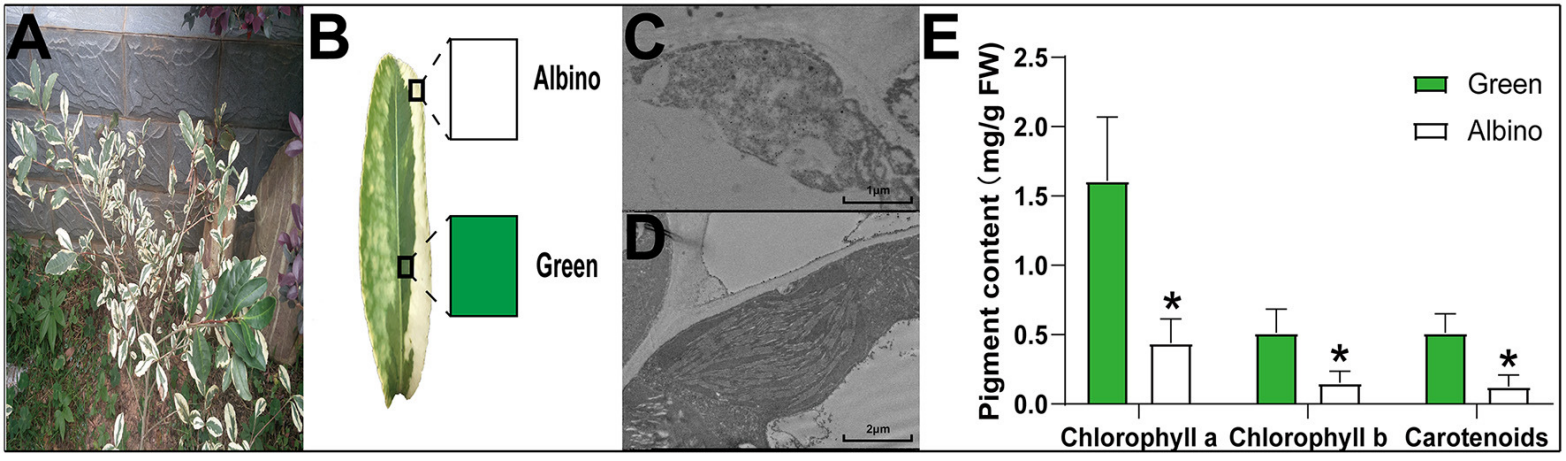
The anatomical characteristics and photosynthetic pigment concentration of ‘Yanlingyinbiancha’. **(A,B)** Phenotypic characteristics of ‘Yanlingyinbiancha’; **(C)** Chloroplast ultrastructure of albino tissues; **(D)** Chloroplast ultrastructure of green tissues; **(E)** Photosynthetic pigments of albino and green tissues. Data were assessed by one-way ANOVA followed by Duncan's multiple range test. * indicates *p* < 0.05.

‘Yanlingyinbiancha’ is a variegated tea plant with stable phenotypic traits, which is not affected by light or temperature. As shown in Figures 1A,B, the leaves of ‘Yanlingyinbiancha’ displayed a variegated phenotype, with green and albino tissues. TEM analysis showed the chloroplasts of albino tissues to have no intact lamellar structure, whereas those of green tissues were fully developed (Figures 1C,D). As shown in Figure 1E, chlorophyll a, chlorophyll b, and carotenoid contents in green tissues were 3.66, 3.48, and 4.05 times higher than in albino tissues (*p* < 0.05). These indicate that chloroplast developmental disorders and a deficiency in chlorophylls and carotenoids are important factors leading to the variegated phenotype of ‘Yanlingyinbiancha’.

### Changes in Catechins, Caffeine, Gallic Acid, and Theobromine Contents

To explore the differences in the biochemical components of albino and green tissues, the contents of catechins (EGC, C, EC, EGCG, GCG, and ECG), caffeine (CAF), gallic acid (GA), and theobromine (TB) in the tested tea leaf samples were determined. Table 1 shows the concentration of nine biochemical components in both the tissues to range from 0.013 ± 0.006% to 2.187 ± 1.795%. TB, CAF and catachins contents in albino tissues were higher than in green tissues, while GA content in albino tissues was lower than in green tissues. Among them, CAF and non-galloylated catechins (EGC, C, and EC) contents in albino tissues were 7.88-fold, 2.62-fold, 2.93-fold, and 2.53-fold higher than in green tissues, respectively (*p* < 0.05, each). Catechins, together with CAF, are mainly responsible for the bitter and astringent taste, and overall quality of the tea (Chaturvedula and Prakash, 2011). Compared with green leaves, albino tea leaves usually contain lower levels of catechins and CAF (Feng et al., 2014). This is contrary to our results and needs to be further explored.

**Table 1:** Biochemical compositions of albino and green tissues.

**Components**	**Green tissues**	**Albino tissues**
TB (%)	0.013 ± 0.006	0.033 ± 0.023
GA (%)	0.063 ± 0.035	0.04 ± 0.01
CAF (%)	0.267 ± 0.129	2.103 ± 0.537^*^
EGC (%)	0.487 ± 0.136	1.277 ± 0.41^*^
C (%)	0.19 ± 0.052	0.557 ± 0.156^*^
EC (%)	0.147 ± 0.04	0.373 ± 0.119^*^
EGCG (%)	1.007 ± 0.356	2.187 ± 1.795
GCG (%)	0.407 ± 0.095	0.77 ± 0.311
ECG (%)	0.717 ± 0.277	1.68 ± 1.207

*EGC, epi-gallocatechin; C, (–)-catechin; EC, epi-catechin; EGCG, epi-gallocatechin gallate; GCG, gallocatechin-3-gallate; ECG, epi-catechin gallate; CAF, caffeine; TB, theobromine. Data were assessed by one-way ANOVA followed by Duncan's multiple range test*.

* *indicates p < 0.05*.

### Transcriptome Analysis

#### *De-novo* Assembly

In total, 41.14 Gb clean data with a Q30 value of 92.27% and GC content of 44.26% were obtained after rigorous quality control and data cleansing, indicating high-quality sequencing results for subsequent analysis (Supplementary Table 2). On an average, 82.43% of clean reads were aligned to *C. sinensis* reference genome (Wei et al., 2018); the mapping statistics are presented in Supplementary Table 3. Uniformity of the mapping result for each sample suggested that they were comparable. A Pearson's correlation coefficient heatmap and principal component analysis between each pair of biological replicates were created (Supplementary Figure 1). Results showed the sequencing data used in the present study to be highly reliable. This dataset has been deposited in NCBI with BioProject number PRJNA733288.

#### GO and KEGG Analysis of Differentially Expressed Genes

**Figure 2:**
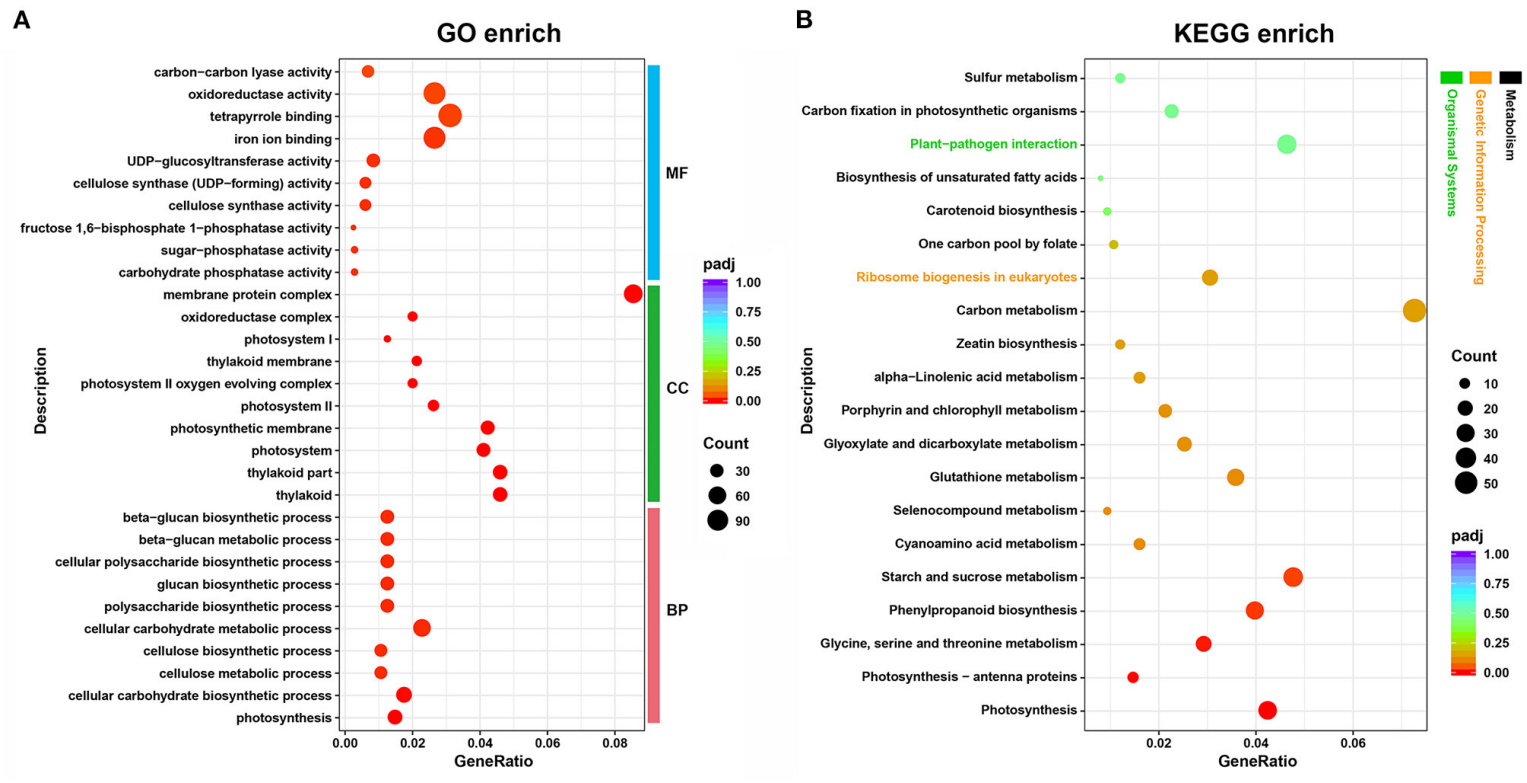
Functional annotation and classification of DEGs identified in the tea plant transcriptome, as determined using the GO and KEGG databases. **(A)** GO enrichment analysis; **(B)** KEGG enrichment analysis. GeneRatio, the ratio between DEGs annotated to a particular KEGG pathway/GO term and all DEGs annotated to KEGG pathway/GO category; padj, *p*-values corrected for multiple hypothesis testing, a lower padj indicates more significant enrichment of the DEGs; Count, the number of DEGs annotated to KEGG pathway/GO term; MF, molecular function; CC, cellular component; BP, biological process.

In total, 7,995 DEGs, including 3,407 upregulated and 4,588 downregulated in albino tissues, were identified (Supplementary Figure 2). As shown in Figure 2, “photosynthesis”, “cellular carbohydrate biosynthetic process”, and “cellular carbohydrate metabolic process” were the most highly enriched GO terms in the biological process (BP) category, whereas “thylakoid”, “thylakoid part”, and “photosystem” were highly enriched in the cellular component (CC) category. GO molecular function (MF) analysis showed “carbohydrate phosphatase activity”, “sugar-phosphatase activity”, “fructose 1,6-bisphosphate 1-phosphatase activity” to be the most enriched terms. In addition, the largest number of DEGs were annotated to tetrapyrrole binding, iron ion binding, and oxidoreductase activity. KEGG enrichment analysis revealed most of the DEGs to be enriched in “carbon metabolism” (55), and “starch and sucrose metabolism” (36). “Photosynthesis”, “photosynthesis-antenna proteins”, “glycine, serine, and threonine metabolism”, “phenylpropanoid biosynthesis”, and “starch and sucrose metabolism” were the most highly enriched terms in KEGG pathway (Figure 2). GO and KEGG enrichment analysis indicate that photosynthesis, energy metabolism, chloroplast development and chlorophyll metabolism in albino tissue were significantly affected compared to green tissues. Therefore, we conducted a further analysis of the transcriptome around the above pathways.

#### Biosynthesis of Photosynthetic Pigments

**Figure 3:**
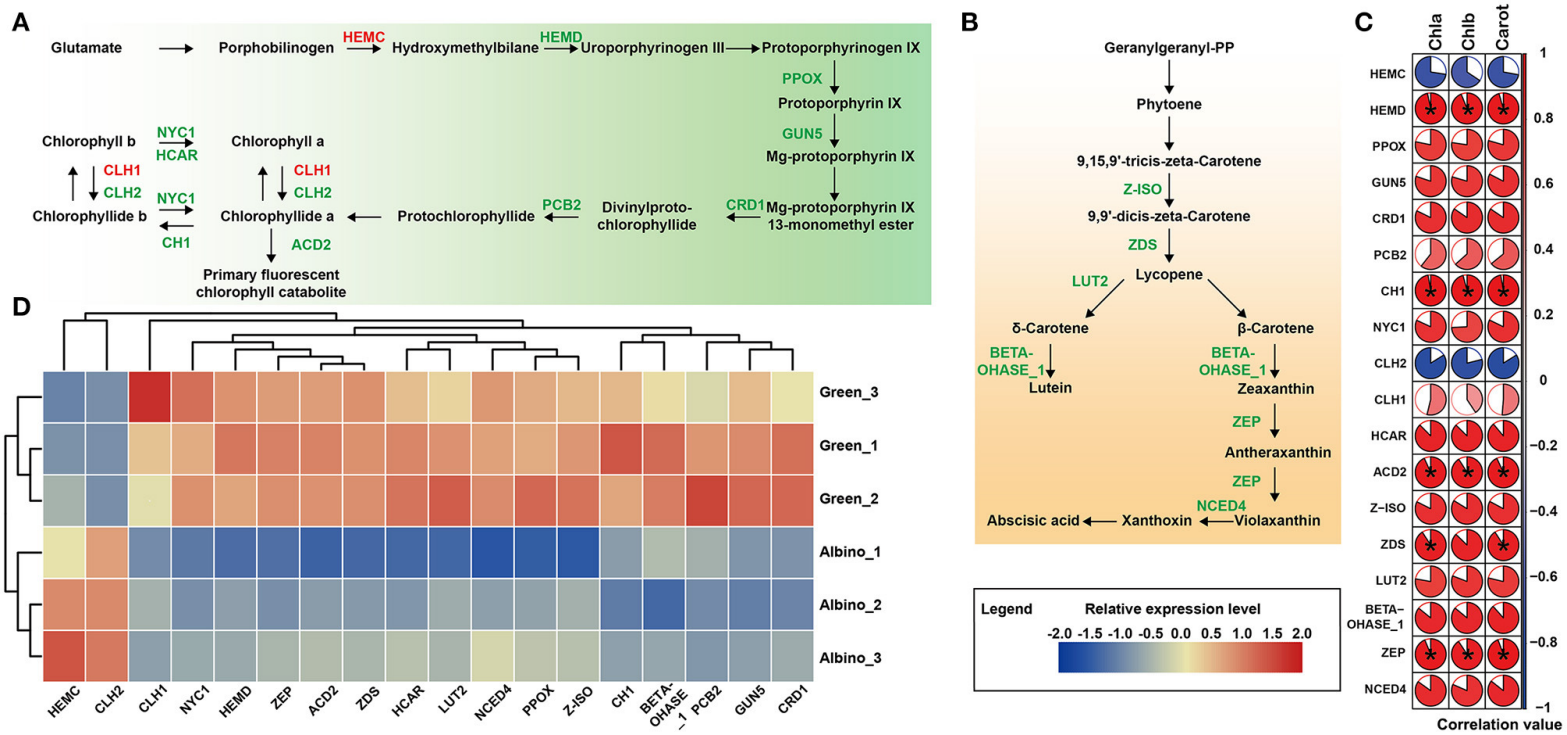
DEGs involved in photosynthetic pigment pathways. **(A)** Chlorophyll biosynthesis; **(B)** Carotenoid biosynthesis (Enzymes in red indicate that the gene encoding the enzyme was upregulated in albino tissue while those in green indicate that the gene encoding the enzyme was downregulated in albino tissue compared to that in green tissues); **(C)** Pearson's correlation analysis between pigments and DEGs (Data were assessed by two-tailed *t*-test. *indicate *p* < 0.05. The area of the white sector indicates the p value of the statistical test of the correlation value); **(D)** Relative expression level of DEGs in both metabolisms. HEMC, hydroxymethylbilane synthase; HEMD, uroporphyrinogen III synthase; PPOX, protoporphyrinogen oxidase; GUN5, magnesium-chelatase subunit chlH; CRD1, magnesium-protoporphyrin IX monomethyl ester cyclase; PCB2, divinyl protochlorophyllide 8-vinyl reductase; NYC1, chlorophyll(ide) b reductase; CH1, chlorophyll a oxygenase; CLH1, chlorophyllase 1; CLH2, chlorophyllase 2; HCAR, 7-hydroxymethyl chlorophyll a (HMChl) reductase; ACD2, red chlorophyll catabolite reductase; Z-ISO, zeta-carotene isomerase; ZDS, zeta-carotene desaturase; LUT2, lycopene epsilon-cyclase; BETA-OHASE_1, beta-hydroxylase 1; ZEP, zeaxanthin epoxidase; NCED4, nine-cis-epoxycarotenoid dioxygenase 4.

We conducted pathway analysis of DEGs involved in chlorophyll and carotenoid metabolism. As shown in Figure 3, most DEGs related to chlorophyll metabolism were downregulated in albino tissues, including six DEGs (*HEMD, PPOX, GUN5, CRD1, PCB2*, and *CH1*) associated with chlorophyll biosynthesis and four DEGs (*NYC1, HCAR, CLH1*, and *ACD2*) mediated chlorophyll degradation. Correlation analysis between pigments and DEGs showed that the expression of *HEMD, CH1*, and *ACD2* gene was significantly correlated with chlorophyll content. All carotenoid synthesis-related DEGs in albino tissues were downregulated, including zeta-carotene isomerase (*Z-ISO*), zeta-carotene desaturase (*ZDS*), lycopene epsilon-cyclase (*LUT2*), beta-hydroxylase 1 (*BETA-OHASE_1*), zeaxanthin epoxidase (*ZEP*), and nine-cis-epoxycarotenoid dioxygenase 4 (*NCED4*) genes. Correlation analysis showed that the expression of *ZDS* and *ZEP* genes was significantly related to carotenoid content. These results showed the decrease of photosynthetic pigments in albino tissues to possibly be due to the deficit in chlorophyll and carotenoid biosynthesis.

#### Photosynthesis

**Figure 4:**
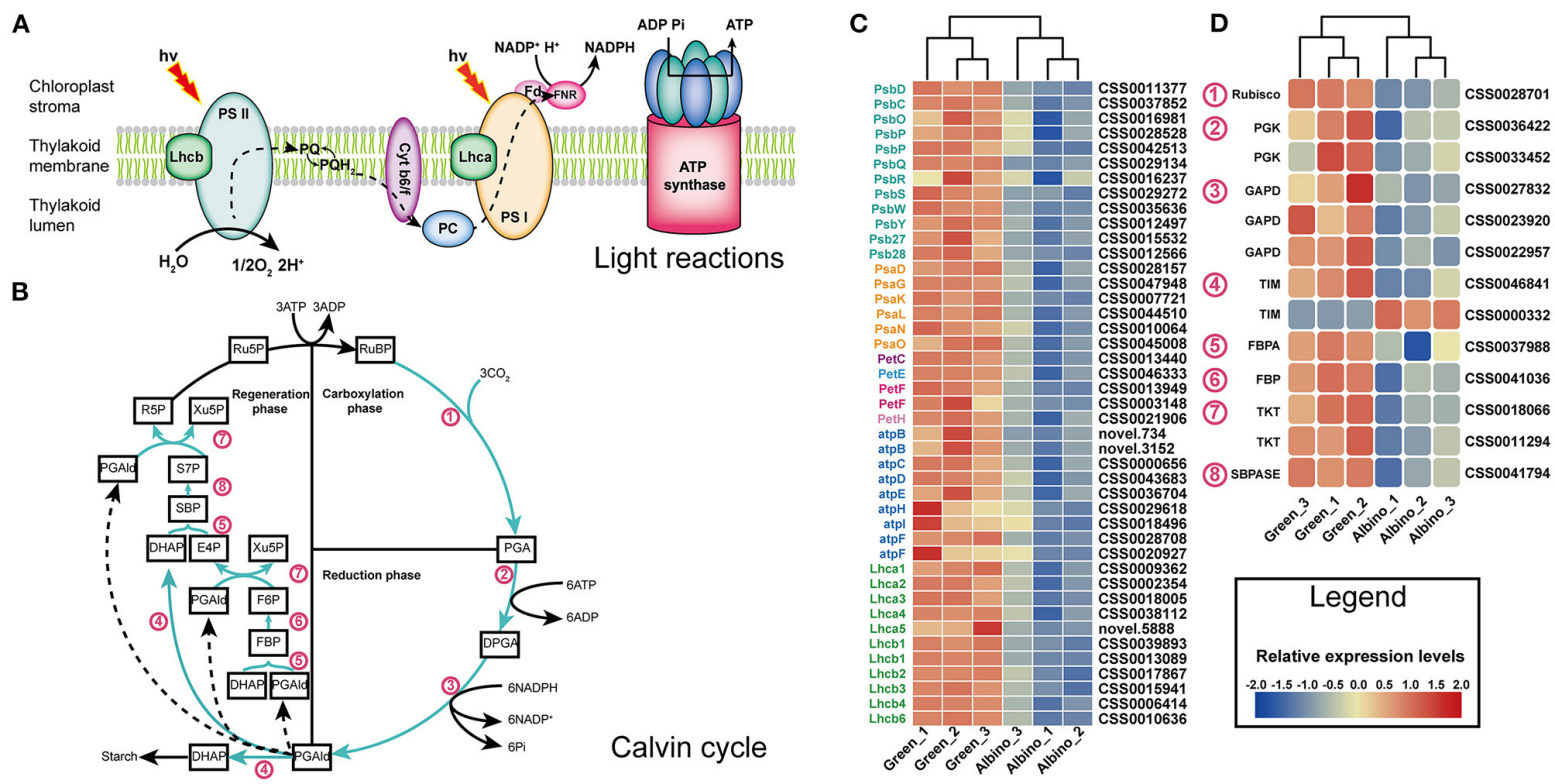
DEGs associated with light reactions **(A)** and Calvin cycle **(B)**. **(C)** Relative expression level of DEGs in light reactions; **(D)** relative expression level of DEGs in Calvin cycle. Lhcb, light harvesting complex of photosystem II; PS II, photosystem II; PQ, plastoquinone; Cyt b6/f, cytochrome b6/f; PC, plastocyanin; Lhca, light harvesting complex of photosystem I; PS I, photosystem I; Fd, ferredoxin; FNR, ferredoxin-NADP+reductase; RuBP, D-Ribulose 1:5-bisphosphate; PGA, 3-Phosphoglycerate; DPGA, 1:3-Bisphospho-D-glycerate; PGAld, Glyceraldehyde 3-phosphate; DHAP, Dihydroxyacetone phosphate; FBP, D-Fructose 1:6-bisphosphate; F6P, D-Fructose 6-phosphate; E4P, D-Erythrose 4-phosphate; Xu5P, D-Xylulose 5-phosphate; SBP, Sedoheptulose 1:7-bisphosphate; S7P, Sedoheptulose 7-phosphate; R5P, Ribose 5-phosphate; Ru5P, Ribulose 5-phosphate; Rubisco, ribulose bisphosphate carboxylase; PGK, phosphoglycerate kinase; GAPDH, glyceraldehyde 3-phosphate dehydrogenase; TIM, triosephosphate isomerase; FBA, fructose-bisphosphate aldolase; FBP, fructose 1,6-bisphosphate 1-phosphatase; TKL, transketolase; SBPASE, sedoheptulose-bisphosphatase.

As shown in Figure 4, all DEGs that mediated the light reactions in albino tissues were downregulated, including twelve genes involved in the photosystem II (PSII) subunit, six in the photosystem I (PSI) subunit, one related to cytochrome b6/f complex, four mediating the photosynthetic electron transport, nine encoding F-type ATPase, and eleven involved in the light-harvesting chlorophyll protein complex (LHC). Except for triosephosphate isomerase gene (*TIM*), all DEGs involved in the Calvin cycle were downregulated. The results indicated that development of chloroplast organization in albino tissues was blocked, which affected photosynthesis remarkably in albino tissues.

#### Energy Metabolism

**Figure 5:**
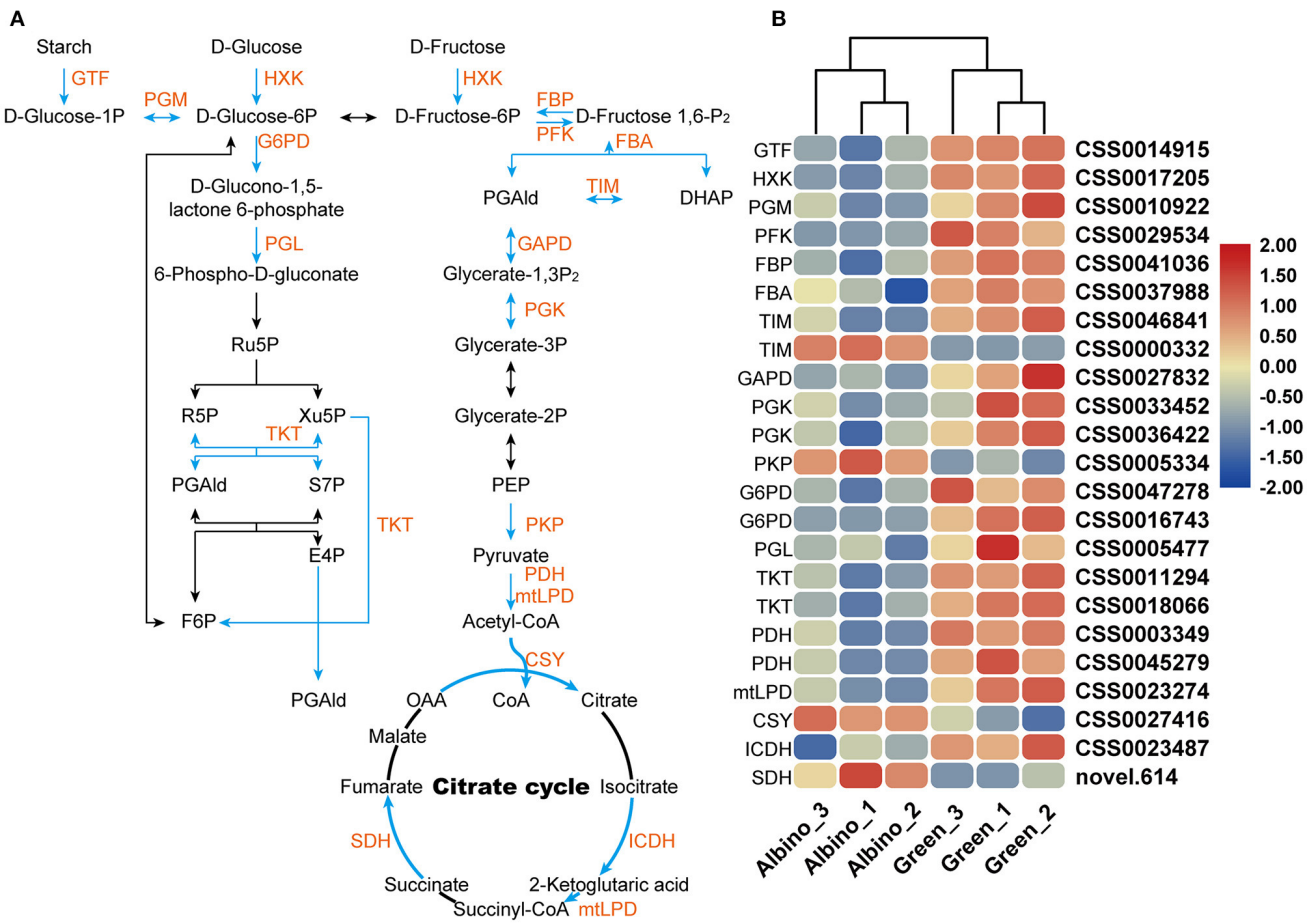
DEGs involved in glycolysis, pentose phosphate pathway, and citrate cycle. **(A)** Energy metabolism; **(B)** relative expression level of DEGs in energy metabolism. GTF, 4-alpha-glucanotransferase; HXK, hexokinase; PGM, phosphoglucomutase; PFK, phosphofructokinase; FBP, fructose 1,6-bisphosphate 1-phosphatase; FBA, fructose-bisphosphate aldolase; TIM, triosephosphate isomerase; GAPDH, glyceraldehyde 3-phosphate dehydrogenase; PGK, phosphoglycerate kinase; PKP, pyruvate kinase;G6PD, glucose-6-phosphate dehydrogenase; PGL, 6-phosphogluconolactonase; TKL, transketolase; PDH, pyruvate dehydrogenase; mtLPD, mitochondrial lipoamide dehydrogenase; CSY, citrate synthase; ICDH, isocitrate dehydrogenase; SDH, succinate dehydrogenase.

As shown in Figure 5, most DEGs involved in energy metabolism in albino tissues, including phosphofructokinase gene (*PFK*), hexokinase gene (*HXK*), glucose-6-phosphate dehydrogenase gene (*G6PD*), and isocitrate dehydrogenase gene (*ICDH*), were downregulated. They encoded key enzymes involved in glycolysis, pentose phosphate pathway, and citrate cycle. Although the genes encoding pyruvate kinase (*PKP*) and citrate synthase (*CSY*), which are key enzymes in glycolysis and citrate cycle, were upregulated, energy metabolism in albino tissues was severely disturbed, and green tissues might supply energy to albino tissues.

#### Flavonoid Metabolism

**Figure 6:**
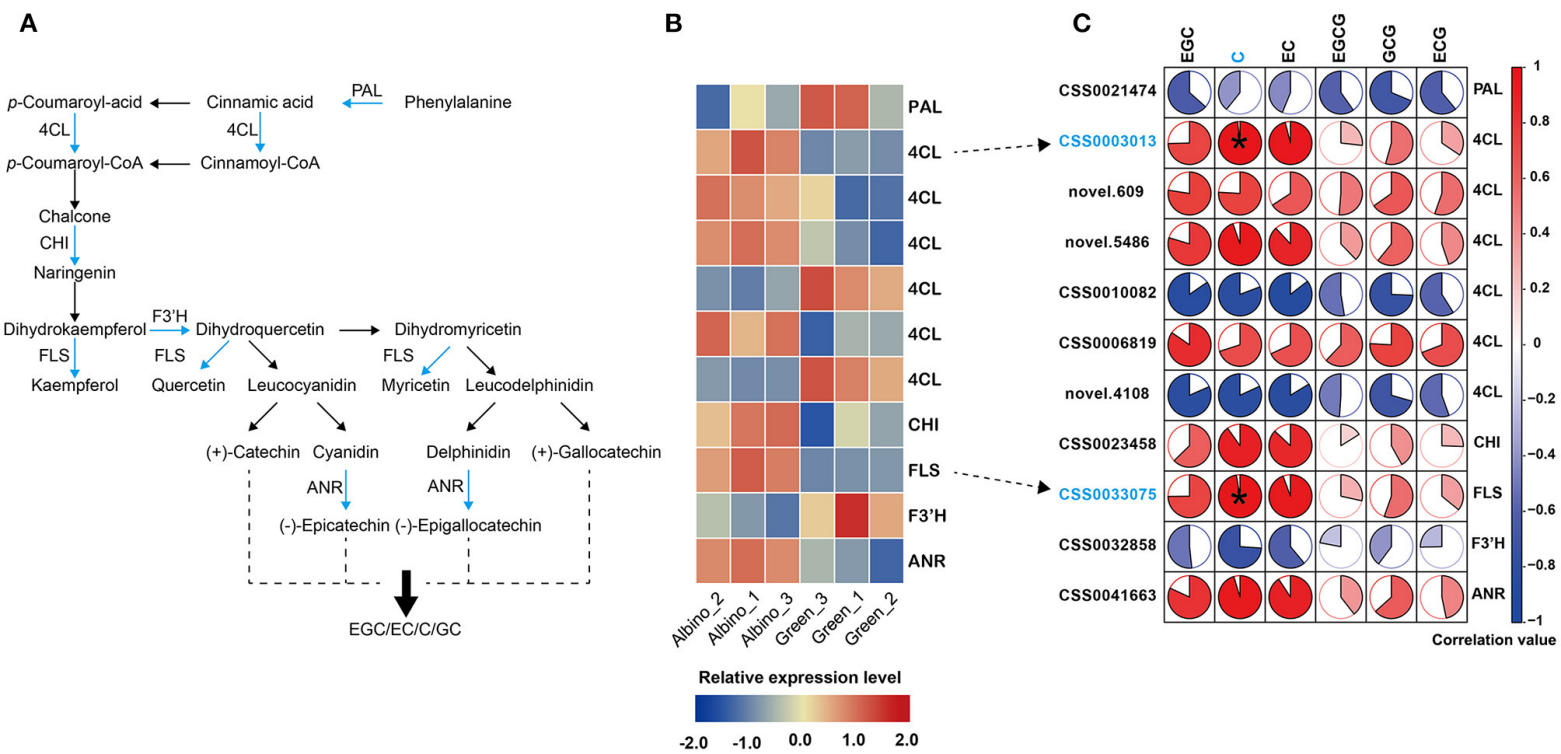
Analysis of flavonoid metabolism. **(A)**: flavonoid biosynthesis; **(B)**: differential expressed genes (DEGs) in different tissues; **(C)**: correlation analysis between catechins and DEGs (Data were assessed by two-tailed *t*-test. *indicate *p* < 0.05. The area of the white sector indicates the p value of the correlation coefficient.). PAL, phenyl alanine ammonia-lyase; 4CL, 4-coumarate: CoA ligase; CHI, chitinase; FLS, flavonol synthase; F3'H, flavanone 3-hydroxylase; ANR, anthocyanidin reductase.

As presented in Figure 6, more than half of the DEGs involved in flavonoid metabolism in albino tissues were upregulated, including four 4-coumarate: CoA ligase (*4CL*) genes, chitinase (*CHI*) gene, flavonol synthase (*FLS*) gene, and anthocyanidin reductase (*ANR*) gene, consistent with the increase in catechin levels in albino tissue. Pearson's correlation analysis showed that *4CL* (CSS0003013) gene and *FLS* gene were positively and significantly correlated with (–)-catechin content. Anthocyanidin reductase converts anthocyanidins to epi-flavan-3-ols (EGC, EC), indicating that the significant increase in EC and EGC contents in albino tissues was mainly attributed to the upregulation of ANR gene. In addition to catechins, the contents of other flavonoids in albino tissues was also likely to increase due to the upregulation of *FLS* gene, such as quercetin, kaempferol and myricetin.

#### Photomorphogenesis

**Figure 7:**
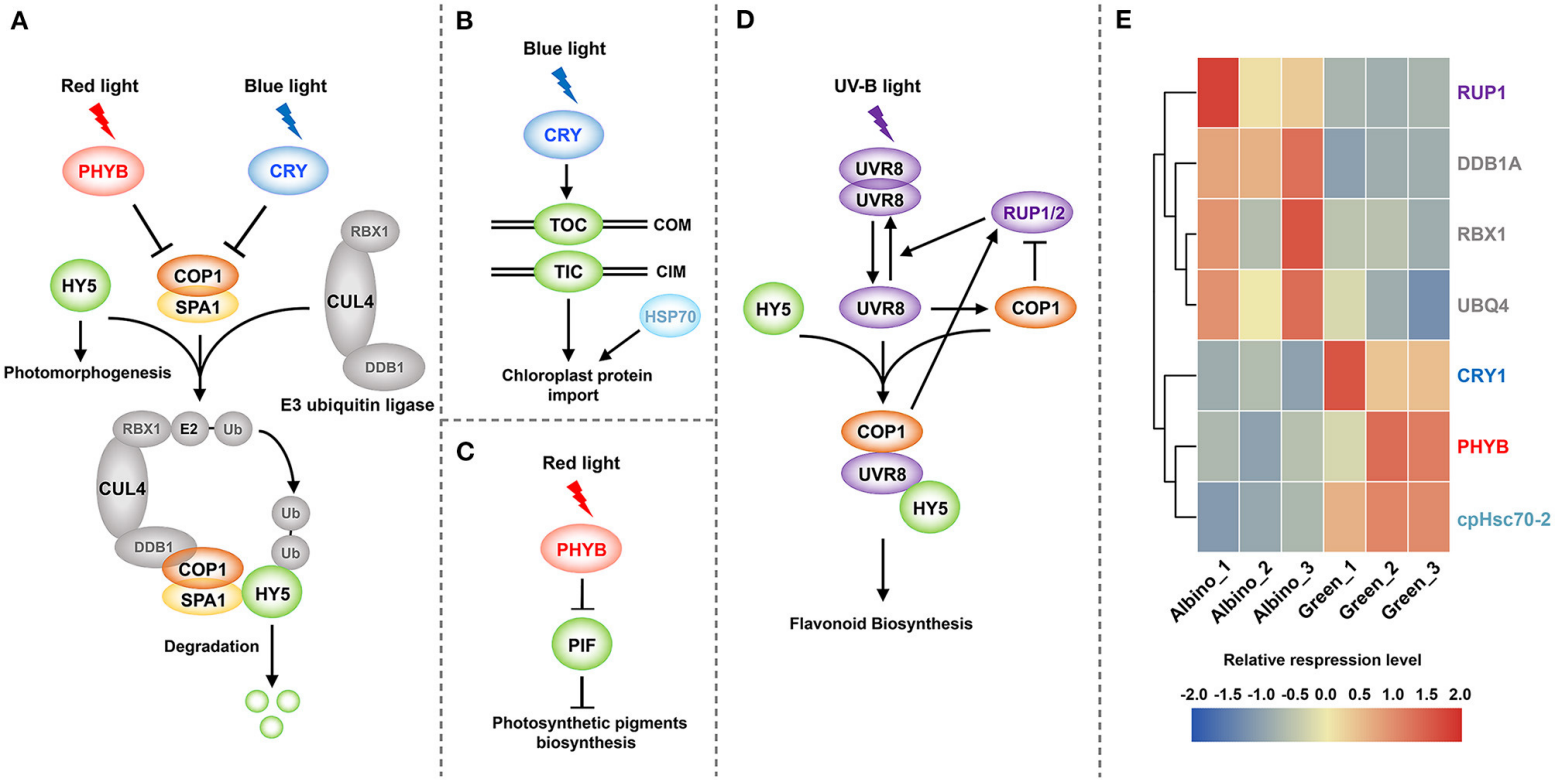
Photomorphogenesis mediates chloroplast development and flavonoid biosynthesis. **(A)** Chloroplast protein-related nuclear gene expression; **(B)** Chloroplast protein import; **(C)**: Photosynthetic pigment biosynthesis; **(D)** UV-B signal mediated flavonoid biosynthesis; **(E)** DEGs involved in photomorphogenesis. PHYB, phytochrome B; CRY, cryptochrome; HY5, ELONGATED HYPOCOTYL 5; COP1, CONSTITUTIVE PHOTOMORPHOGENIC 1; SPA1, SUPPRESSOR OF PHYA-105 1; RBX1, RING-box 1; CUL4, cullin4; DDB1, damaged DNA binding protein 1; Ub, ubiquitin; TOC, translocon at the outer envelope membrane of chloroplasts; TIC, translocon at the inner envelope membrane of chloroplasts; COM, chloroplast outer membrane; CIM, chloroplast inner membrane; HSP70, heat shock protein 70; PIF, phytochrome interacting factor; UVR8, UV RESISTANCE LOCUS 8; RUP1/2, REPRESSOR OF UV-B PHOTOMORPHOGENESIS 1/2.

As presented in Figure 7, DEGs encoding positive regulators of photomorphogenesis involved in chloroplast development were all downregulated in albino tissues, including cryptochrome 1 (*CRY1*), phytochrome B (*PHYB*), and chloroplast heat shock protein 70-2 (*cpHsc70-2*) genes. As shown in Figures 7A–C, *CRY1* and *PHYB* genes encode blue and red light photoreceptors, respectively, and mediate chloroplast nuclear gene expression, chloroplast protein import and photosynthetic pigment biosynthesis gene expression. DEGs participating in protein ubiquitination and degradation were all upregulated, including RING-box 1 (*RBX1*), damaged DNA binding protein 1A (*DDB1A*), and ubiquitin 4 (*UBQ4*) genes. These genes can participate in the degradation of positive regulators of photomorphogenesis. In addition, REPRESSOR OF UV-B PHOTOMORPHOGENESIS 1 (*RUP1*) gene that mediates UV-B signaling pathway was upregulated. UV-B signaling pathway is closely related to flavonoid metabolism, and UV-B signal can promote flavonoid biosynthesis through transcriptional regulation.

### Validation of DEG Transcript Levels

qRT-PCR was used for verification of the RNA-seq results (Supplementary Figure 3). Sixteen DEGs were randomly selected for qRT-PCR verification. Of them, four were involved in chlorophyll biosynthesis (*HEMD, CH1, NYC1*, and *ACD2*), two were involved in carotenoid biosynthesis (*ZDS* and *ZEP*), four mediated the light reaction (*Lhca1, Lhcb1, PsbS*, and *PsbY*), two were related to Calvin cycle (*Rubisco* and *PGK*), three were involved in energy metabolism (*GTF, FBA*, and *PGK*), and two were involved in flavonoid metabolism (*PAL* and *FLS*). Expression pattern of these genes was consistent with RNA-seq data, indicating that the RNA-seq data are reliable.

## Discussion

### Repression of Photosynthetic Pigment Biosynthesis Leads to Albinism

Chlorophyll metabolism, including its biosynthesis, cycling, and degradation, is a complex biological process in plants. In our study, six (*HEMD, PPOX, GUN5, CRD1, PCB2*, and *CH1*) and four (*NYC1, HCAR, CLH1*, and *ACD2*) DEGs associated with chlorophyll biosynthesis and chlorophyll degradation, respectively, were found to be downregulated. Previous work had shown that mutation in the *GUN5* genecan cause the formation of albinos (Mochizuki et al., 2001). Plants with reduced CHL27/CRD1 display chlorosis and excessive MgProtoME accumulation (Tottey et al., 2003; Bang et al., 2008; Peter et al., 2010). Moreover, chlorophyll content of *PCB2* mutant decreased significantly compared to that in wild-type (Nakanishi et al., 2005). Therefore, we considered variegated phenotype to possibly arise from impaired chlorophyll biosynthesis in albino tissues, and the DEGs involved in chlorophyll degradation to possibly be downregulated due to the substrate inhibition effect.

In plants, carotenoids serve as accessory pigments in photosynthesis and protect against photooxidative stress (Britton et al., 2004). Previous studies had shown that plants deficient in genes encoding carotenoid biosynthesis pathway enzymes exhibit an albino phenotype. For example, Chen et al. had reported that Arabidopsis *Z-ISO* mutant plants contained lower levels of carotenoids and chlorophylls than the wild type (Chen et al., 2010). Furthermore, in cases of ZDS deficiency, maize showed defective chloroplast development and reduced photosynthetic pigments, leading to an albino phenotype (Wang et al., 2020). In this study, five DEGs involved in carotenoid biosynthesis were downregulated in albino tissues, indicating the decrease in carotenoid to be due to the reduction in carotenoid biosynthesis. Carotenoids contribute to the maintenance of chlorophyll levels (Wang et al., 2014; Shen et al., 2018); hence, inhibition of carotenoid biosynthesis in albino tissues might be one of the reasons for reduction of chlorophyll and variegated phenotype.

### Photomorphogenesis Affects Chloroplast Development

Chloroplast is essential for photosynthesis as well as the production of hormones and metabolites (Pogson and Albrecht, 2011). Our study found that chloroplasts have no intact lamellar structure in albino tissues, and that photosynthesis was suppressed. Similar results were also observed in other albino or variegated plants (Sun et al., 2017; Shih et al., 2019; Li et al., 2020). As shown in Figure 4A, four major photosynthetic complexes in plants, including PSII, PSI, the cytochrome *b*_6_*/f* (cyt*b*_6_*/f*) complex, and plastid ATP synthase, are located in the chloroplast thylakoid membrane (Rochaix, 2004). These components convert water into oxygen and produce NADPH and ATP. Subsequently, NADPH and ATP are used for CO_2_ assimilation by the Calvin cycle (Fromme et al., 2001; Rochaix, 2011; Allahverdiyeva et al., 2013). In this study, fifty-five DEGs involved in the above processes were downregulated in albino tissues, indicating the light reaction to be severely suppressed and Calvin cycle to be inhibited by the shortage of ATP and NAPDH.

One major environmental factor determining the biogenesis of chloroplast is light, which acts via photomorphogenic pathways to control the transcription of genes encoding chloroplast-targeted protein (Pogson and Albrecht, 2011). The perception of light has been shown to require the activation of photoreceptors, such as phytochromes (PHYs) and cryptochromes (CRYs). As shown in Figure 7A, phytochromes and cryptochromes inhibit the function of COP1 protein upon irradiation of red and blue light. In the dark, COP1-SPA1 complex can combine with E3 ubiquitin ligase to degrade ELONGATED HYPOCOTYL 5 (HY5) transcription factor, and thereby inhibits photomorphogenesis. In our study, *PHYB* and *CRY1* genes were dramatically downregulated in albino tissues. Moreover, based on the observation that *RBX1, DDB1A*, and *UBQ4* genes that encode E3 ubiquitin ligase were upregulated in albino tissues, we speculated that the destruction of photoreceptors in albino tissues causes COP1 to accumulate in the nucleus. Subsequently, COP1 mediates the degradation and ubiquitination of positive transcription factors of photomorphogenesis and prevents light-induced gene expression. These results show that downregulation of *PHYB* and *CRY1* genes in albino tissues might lead to downregulation of the gene encoding chloroplast protein that is involved in the light reaction.

In addition to control the transcription of genes encoding chloroplast protein, light can also affect the process of protein import into chloroplast stroma. Previous study had shown that induction of translocon at the outer envelope membrane of chloroplasts (*TOC*) and translocon at the inner envelope membrane of chloroplasts (*TIC*) genes, which encode components of the chloroplast protein import apparatus, is mediated by *CRY1* (Fukazawa et al., 2020). This suggested that downregulation of *CRY1* in albino tissues might inhibit the expression of *TOC* and *TIC* genes, leading to a blockage in chloroplast protein import. Moreover, we also observed the downregulation of *cpHsc70-2* gene. Previous study had shown that *cpHsc70-2* gene is important for protein import into chloroplasts (Su and Li, 2010). Hence, we considered the disruption of chloroplast protein import pathway in albino tissues to be one of the reasons for abnormal chloroplast development.

Chlorophylls and carotenoids are essential for the assembly and protection of photosynthetic apparatus. PHYB, as the predominant member of PHY family, promotes the ubiquitination and degradation of phytochrome interacting factors (PIFs) (Leivar et al., 2012). PIFs are negative regulators of tetrapyrrole biosynthesis genes and carotenoid biosynthesis genes (Gommers and Monte, 2018; Xu, 2020). As shown in Figure 7C, downregulation of PHYB might lead to the accumulation of PIFs in albino tissues, and thus inhibited the biosynthesis of photosynthetic pigment. Deficit in chlorophylls and carotenoids affected the assembly and maintenance of photosynthetic apparatus, which is highly likely another reason for abnormal chloroplast development.

Taken together, as presented in Figures 7A–C, we conjectured that the destruction of photoreceptors suppressed photomorphogenesis in albino tissues, leading to the inhibition of chloroplast protein gene transcription, blockage of chloroplast protein import, and damage to the assembly and protection of photosynthetic apparatus. These factors together prevented the development of chloroplasts in albino tissues, resulting in a weakened capacity for photosynthesis.

### Survival Strategy of Albino Tissues Under Carbon Starvation Condition

Considering the inhibition of photosynthesis due to defects in chloroplast development, carbon supply for the survival of albino tissues is insufficient. Therefore, we speculated that the albino tissues implemented three strategies to compensate for this deficit. First, albino tissues may require sucrose delivery from green tissues. Sucrose synthase (*SUS*) has long been considered a biochemical marker for sink strength (Xu et al., 2012). It catalyzes the hydrolysis of sucrose to UDP-glucose and fructose, thus forming a gradient that promotes sucrose transport. Upregulation of SUS gene was observed in albino tissues, so we speculated that albino tissues may receive sucrose from green tissues, as a sink tissue. The same conclusion has been obtained in the study of variegated mutant in *Arabidopsis* (Aluru et al., 2009). Second, fatty acid respiration is probably a backup mechanism under energy shortage conditions (Kunz et al., 2009). The DEGs involved in β-oxidation of fatty acid in albino tissues, including long-chain acyl-CoA synthetase (*LACS*), acyl-CoA oxidase (*ACX*) and peroxisomal 3-ketoacyl-CoA thiolase (*PKT*) genes, were upregulated. Our results suggested that the albino tissues treat fatty acid oxidation as energy supplement under carbon starvation conditions induced by the inhibition of photosynthesis. Third, anaerobic respiration in albino tissues might increase to compensate for the repression of mitochondrial respiration. Aldehyde dehydrogenase 2 (*ALDH2*) participates in the detoxification process of alcohol fermentation (Kirch et al., 2004). We found *ALDH2B4* and *ALDH2B7* genes to be significantly upregulated in albino tissues, in agreement with our speculation.

### UV-B Signal and Brassinosteroids Mediate Flavonoid Biosynthesis

The higher content of catechins in albino tissues may result from two reasons. Previous study had shown that the expression of flavonoid pathway genes, such as *FLS, 4CL*, and *ANR*, could be induced in response to UV-B signal, leading to the accumulation of catechins in *Camellia sinensis* (Fan et al., 2018; Liu et al., 2018). While COP1 is a negative regulator of photomorphogenesis, it acts as a positive regulator in the UV-B signaling pathway (Jin and Zhu, 2019). The accumulation of COP1 in albino tissues may promote UV-B signaling and thus increase the level of catechins. As presented in Figure 7D, *RUP1/2* were transcriptionally activated by UV-B in a COP1, UVR8, and HY5-dependent manner, and negatively regulated the UV-B pathway to prevent overstimulation by UV-B (Gruber et al., 2010). The upregulation of *RUP1* indirectly proved the UV-B signaling pathway to possibly be activated by COP1. Moreover, upregulation of DEGs involved in brassinosteroid (BR) biosynthesis and signal transduction, such as brassinosteroid-6-oxidase 2 (*BR6OX2*), CONSTITUTIVE PHOTOMORPHOGENIC DWARF (*CPD*), and BR-signaling kinase 2 (*BSK2*) genes, was observed. BR can upregulate the expression of genes involved in catechin synthesis by increasing the accumulation of nitric oxide (NO) (Li et al., 2016b, 2017). Our results suggested that the increase in endogenous BR levels might lead to the higher content of catechins in albino tissues.

## Conclusion

‘Yanlingyinbiancha’ has a variegated phenotype owing to the lack of pigment on the edge of the leaves. Analysis of the transcriptome, in this study, showed that chloroplast development in albino tissues might be repressed by three factors: inhibition of chloroplast protein gene transcription, blockage of chloroplast protein import, and damage of the assembly and protection of photosynthetic apparatus. Abnormal chloroplast development inevitably leads to the destruction of photosynthesis and insufficient energy. Therefore, the albino tissues might receive sucrose from the green tissues and decompose their own storage substances to obtain the energy needed for survival. Further, catechins, as a characteristic secondary metabolite of tea plants, accumulated in albino tissues through UV-B signals and hormone signals. Variegated mutants are powerful tools for understanding mechanisms of chloroplast biogenesis. This study preliminarily reveals the difference between albino and green tissues of ‘Yanlingyinbiancha’ and the molecular mechanism underlying variegation.

## Data Availability Statement

The original contributions presented in the study are publicly available. This data can be found here: NCBI repository, accession number: PRJNA733288.

## Author Contributions

XG: conceptualization, writing—original draft, and mapping. CZ: writing—review and editing and mapping. CL: investigation, resources, and supervision. MW and NX: validation. JianC: formal analysis. YL: data curation. JiaC: visualization. CS: project administration and funding acquisition. All authors contributed to the article and approved the submitted version.

## Funding

This work was supported by Joint Funds of National Natural Science Foundation of China (grant number U19A2030); the National Natural Science Foundation of China (grant number 31271789); Central Committee Guides Local Science and Technology Development Program (grant number 2019XF5041); and the Special Project for the Construction of Modern Agricultural Industrial Technology Systems in Hunan Province (Xiangcai Nongzhi) (grant number 2020112).

## Conflict of Interest

The authors declare that the research was conducted in the absence of any commercial or financial relationships that could be construed as a potential conflict of interest.

## Publisher's Note

All claims expressed in this article are solely those of the authors and do not necessarily represent those of their affiliated organizations, or those of the publisher, the editors and the reviewers. Any product that may be evaluated in this article, or claim that may be made by its manufacturer, is not guaranteed or endorsed by the publisher.
