# Supplementary material for: Disruption of Photomorphogenesis Leads to Abnormal Chloroplast Development and Leaf Variegation in Camellia sinensis
Source: Front Plant Sci. 2021 Sep 9;12:720800. doi: 10.3389/fpls.2021.720800 (PMC8459013; doi:10.3389/fpls.2021.720800)
Supplement: Supplementary file 1 [file Data_Sheet_1.docx]

Supplementary Material

# Supplementary Figures and Tables

**Supplementary Table 1.** Primer information

| Primer name | Sequence |
| --- | --- |
| β-actin-F | TGCCTGCCATGTATGTTGCCATC |
| β-actin-R | GCTCACACCATCACCCGAATCC |
| HEMD-F | CTTCGTCTTCGTCTTCGGTGTCTG |
| HEMD-R | CATTGATGAGCTTGCCGTTCTTGC |
| CH1-F | ACGCTATGTCTGCCATTGCTACAG |
| CH1-R | TGTCTACTAACCCGCCTTCTTCCC |
| NYC1-F | CGCAGTTCCGAGTCTGTGGATATG |
| NYC1-R | GTGCCAGATTTGTCCCAGATGAGG |
| ACD2-F | GCGGAACAGAGCGTATGGAAGAG |
| ACD2-R | CCCTGTCAGCCTCTCCCATATCTC |
| ZDS-F | GGGAGGAATTGGTGGCATTACGG |
| ZDS-R | TGCTTGCACGGTGGTCTATGAATG |
| ZEP-F | TTGGAGGTTGCTGACGAGGTTATG |
| ZEP-R | CCGCCGCAGGAGTGAATGTATC |
| Lhca1-F | TCTGCCCGTCAGTCCTTTCCTC |
| Lhca1-R | TGCGGAGCCGTCAAGATAGGG |
| Lhcb1-F | GTCAAGTTCGGCGAGGCTGTG |
| Lhcb1-R | CTTTGGGCATGGATCAAGCTAGGG |
| PsbS-F | TCGGCTTTGCTGCATCTCTGTTG |
| PsbS-R | GCTCCAATGGCTCCAAGAAGGG |
| PsbY-F | TGGCAGCAACTATAGCAACGATGG |
| PsbY-R | TGGGAGGTTTTGGATGGAGAGGAG |
| Rubisco-F | AGGGTACTACGATGGGCGATACTG |
| Rubisco-R | GCACTCCTCAACCTCCTTCAACAC |
| GTF-F | TTCTGAGATGGGAGGCTGGGATG |
| GTF-R | CCAATCTGCCAGAGGTCACGAAG |
| FBA-F | TGGCGTACTGTTGTGAGCATTCC |
| FBA-R | GGCAGCGTAGCGAGCAAGAC |
| PGK-F | GCCGCTGACCCTCTTCTCTCC |
| PGK-R | AGGTCTCCGACGCTCTTCTTGG |
| PAL-F | GTCGGTTCTGGTCTCGCTTCTATG |
| PAL-R | GCCTGGATGGTGCTTCAATTTGTG |
| FLS-F | AGAGAGTGCAAGCCCTGTCC |
| FLS-R | AGCCTTGCTGTTCTCCGGTT |

**Supplementary** **Table 2.** Summary of sequencing reads.

| Sample | Raw reads | Clean reads | Clean bases | Error rate | Q20 (%) | Q30 (%) | GC (%) |
| --- | --- | --- | --- | --- | --- | --- | --- |
| A_1 | 45,210,640 | 44,581,778 | 6.69G | 0.02 | 96.69 | 91.92 | 43.80 |
| A_2 | 47,700,262 | 47,125,502 | 7.07G | 0.01 | 96.86 | 92.29 | 43.74 |
| A_3 | 46,145,886 | 45,197,934 | 6.78G | 0.02 | 96.73 | 92.01 | 44.36 |
| G_1 | 46,143,060 | 45,235,840 | 6.79G | 0.01 | 96.92 | 92.44 | 44.99 |
| G_2 | 45,908,698 | 45,091,318 | 6.76G | 0.01 | 96.94 | 92.43 | 44.15 |
| G_3 | 47,781,320 | 46,997,528 | 7.05G | 0.01 | 96.99 | 92.52 | 44.51 |

**Supplementary Table 3.** Mapping statistics.

| sample | Total reads | Total map | Unique map | Multi map | Proper map |
| --- | --- | --- | --- | --- | --- |
| A_1 | 44,581,778 | 36,757,449 (82.45%) | 31,567,798 (70.81%) | 5,189,651 (11.64%) | 28,258,982 (63.39%) |
| A_2 | 47,125,502 | 38,953,632 (82.66%) | 33,213,118 (70.48%) | 5,740,514 (12.18%) | 29,851,958 (63.35%) |
| A_3 | 45,197,934 | 37,533,784 (83.04%) | 32,057,956 (70.93%) | 5,475,828 (12.12%) | 28,911,934 (63.97%) |
| G_1 | 45,235,840 | 36,594,605 (80.9%) | 31,196,110 (68.96%) | 5,398,495 (11.93%) | 28,336,056 (62.64%) |
| G_2 | 45,091,318 | 37,555,558 (83.29%) | 32,107,000 (71.2%) | 5,448,558 (12.08%) | 29,070,938 (64.47%) |
| G_3 | 46,997,528 | 38,657,507 (82.25%) | 33,126,466 (70.49%) | 5,531,041 (11.77%) | 30,147,644 (64.15%) |


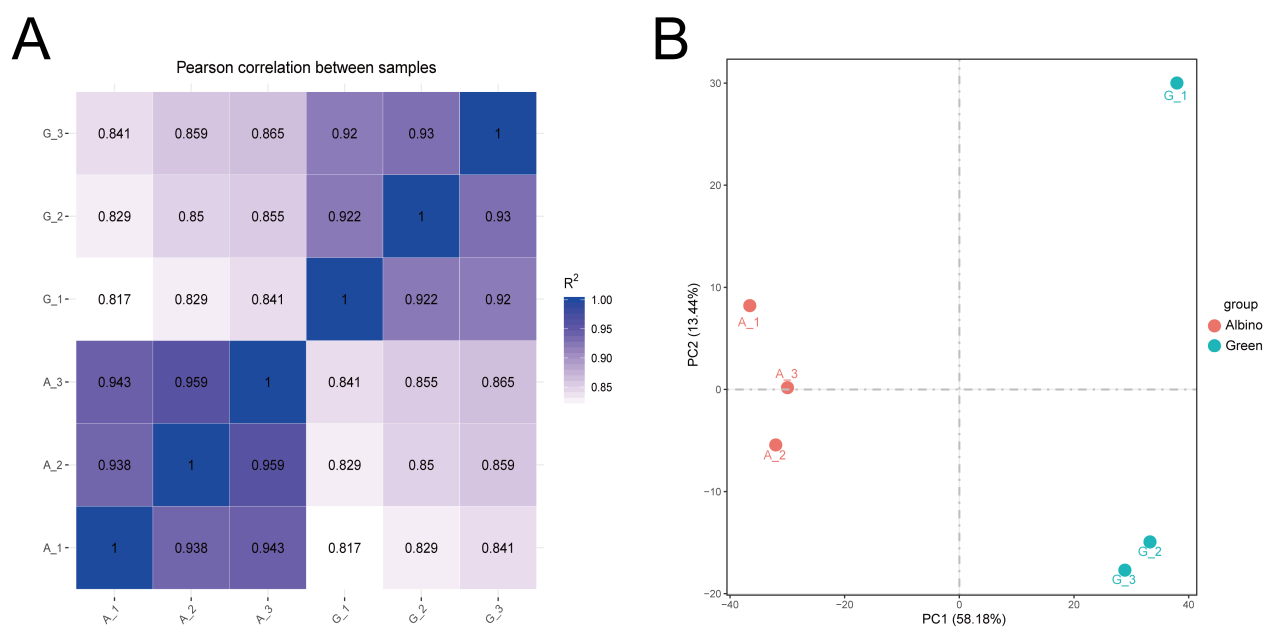


**Supplementary Figure 1.** Inter group analysis. A: Pearson correlation; B: PCA analysis.


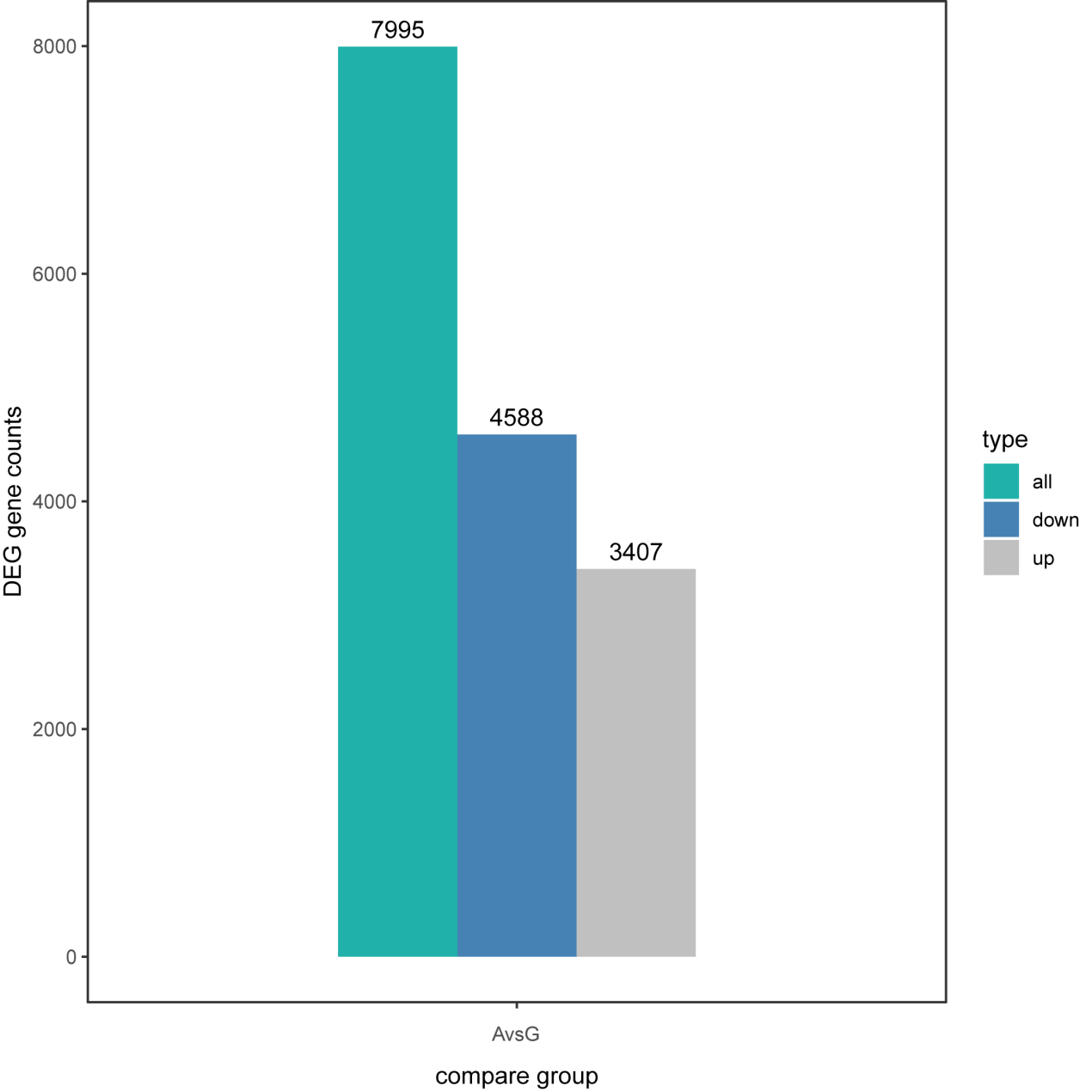


**Supplementary Figure 2.** Numbers of differentially expressed genes (DEGs); down – downregulated DEGs; up – upregulated DEGs.





**Supplementary Figure 3.** qRT-PCR-based verification of the DEGs.
